# *Cornus officinalis* prior and post-processing: Regulatory effects on intestinal flora of diabetic nephropathy rats

**DOI:** 10.3389/fphar.2022.1039711

**Published:** 2022-10-07

**Authors:** Cheng-Guo Ju, Lin Zhu, Wei Wang, Hui Gao, Yu-Bin Xu, Tian-Zhu Jia

**Affiliations:** ^1^ College of Pharmacy, Liaoning University of Traditional Chinese Medicine, Dalian, China; ^2^ Beijing Jujing Health Technology Group, Beijing, China

**Keywords:** cornus officinalis, wine cornus officinalis, wine honey cornus officinalis, DN, Wnt/β-catenin signaling pathway, intestinal flora

## Abstract

**Background:** Diabetic nephropathy (DN) is one of the most common and serious chronic complications in the clinic. *Cornus officinalis* has the effects of replenishing *qi* and nourishing *yin*, tonifying liver and kidney, and it is one of the main traditional Chinese medicines used clinically to treat diabetes and its complications. However, the effect and mechanism of *Cornus officinalis* before and after processing on intestinal flora of diabetic nephropathy need to be further elucidated.

**Methods:** SD rats were randomly divided into a blank group (10 rats) and DN groups (70 rats). After 4 weeks of high-sugar and high-fat diet, the DN rat model was established by intraperitoneal injection of streptozotocin. After successful modeling, the rats were randomly divided into DN model group, irbesartan group (1.35 mg·kg^−1^), *Cornus officinalis* group (281.25 mg·kg^−1^), wine Cornus officinalis group (281.25 mg·kg^−1^), wine honey Cornus officinalis group (281.25 mg·kg^−1^), auxiliary wine group (10 ml·kg^−1^), auxiliary wine honey group (10 ml·kg^−1^). During the observation of the rats’ general state, after 6 weeks of continuous administration, the fasting blood glucose of rats in each group was detected, and the kidney index was calculated. The serum creatinine levels, urea nitrogen and 24 h urinary microalbumin were detected by enzyme-linked immunosorbent assay. The expression levels of YKL-40, Wnt4, *β*-catenin and TGF-*β*_1_ mRNA in renal tissue were detected by fluorescence quantitative PCR. Hematoxylin-eosin staining was used to observe the changes in renal pathological injury in each group; GC-MS detected the changes of short chain fatty acid content. Feces were collected for 16 s high-throughput sequencing to analyze the effects of *Cornus officinalis* on the diversity of intestinal flora in DN before and after processing.

**Results:** Compared with the blank group, the serum creatinine, urea nitrogen, 24 h urinary microalbumin, kidney index and fasting blood glucose in the DN model group were significantly increased (*p* < 0.05). The renal tissue morphology was disordered and a large number of inflammatory cells were infiltrated. The expression of YKL-40, Wnt4, *β*-catenin and TGF-*β*_1_ mRNA was significantly increased (*p* < 0.05). Compared with the DN model group, the serum creatinine, urea. Nitrogen, 24 h urine microalbumin, kidney index and fasting blood glucose of rats in each administration group were significantly decreased (*p* < 0.05), and the general condition and pathological renal damage of DN rats were improved. The effect of wine honey *Cornus officinalis* was the best, and the expression of YKL-40, Wnt4, *β*-catenin and TGF-*β*_1_ mRNA was significantly decreased (*p* < 0.05). In each administration group, the improvement of the above indicators in the wine honey Cornus officinalis group was significantly better than that in the raw Cornus officinalis group and wine Cornus officinalis group (*p* < 0.05), There was no significant difference compared with the irbesartan group (*p* > 0.05). Each administration group had a significant callback effect on the content of short-chain fatty acids in rat feces, with increased intestinal beneficial bacteria and decreased pathogenic bacteria. Compared with the blank group, the abundance of Firmicutes in the DN model group increased, the abundance of Bacteroidetes decreased, and the ratio showed an upward trend in the DN model group decreased. Each administration group could improve the relative abundance of the above intestinal flora in the model group to varying degrees.

**Conclusion:** The processing of *Cornus officinalis* may improve the renal injury of DN rats by blocking the activation of Wnt/β-catenin signaling pathway, regulating the structural composition of intestinal microorganisms, and ultimately playing a role in renal protection.

## Introduction

Diabetic nephropathy (DN) is one of the most serious and common chronic complications of diabetes mellitus, It is one of the main causes of end-stage renal disease and an important factor in the death of diabetic patients, causing serious harm to human health (Sayed et al., 2012; Yang et al., 2020). So far, the pathogenesis of DN has not been elucidated. Existing research data show that abnormal glucose metabolism, inflammatory response, oxidative stress, endoplasmic reticulum stress, autophagy, and exosomes can cause DN (Shikata and Shikata, 2013; Li et al., 2019). At present, western medicine mainly prevents the occurrence and deterioration of DN by controlling blood pressure, controlling blood lipid and anti-inflammation. Traditional Chinese medicine mainly through oral Chinese medicine, acupuncture, enema treatment, but failed to fully elucidate the pathogenesis of DN and the treatment of specific drugs (Liu et al., 2019; Feng, 2022). Therefore, active prevention and treatment of DN and inhibition of the development of the disease are the main means to avoid renal failure and prolong the life of patients.

*Cornus officinalis* Siebold & Zucc. Cornaceae. (*Cornus officinalis*), alias *Cornus officinalis*, Shuzao, Shizao, Rouzao, etc., is a plant of Cornus genus of Cornus family. It is a perennial small tree or shrub. It is traditionally used as a traditional Chinese medicine to remove the dry and mature pulp of the fruit core. It has the effect of tonifying the liver and kidney, astringing the essence and removing the essence. It is mainly used for treating vertigo tinnitus, lumbar and knee pain, impotence and spermatorrhea, internal heat and diabetes (Yang et al., 2016; Lou et al., 2021). Studies have shown that *Cornus officinalis* has anti-tumor, myocardial protection, treatment of diabetes and its complications, hypoglycemic, liver and kidney protection, anti-inflammatory and other pharmacological effects (Jin and Chang, 2015; Zhou et al., 2020). Iridoid glycosides of *Cornus officinalis* is one of the effective components of *Cornus officinalis*. Its characteristic components loganin and morroniside can significantly inhibit the proliferation of renal cortical endothelial cells in DN rats and protect the integrity of endothelium (Yang et al., 2019). Clinical studies have shown that *Cornus officinalis* can effectively improve insulin resistance, reduce blood glucose, and improve the symptoms of diabetic nephropathy. (Park et al., 2013; Qi et al., 2014). Our previous study found that wine honey processed *Cornus officinalis* can improve the sour taste of *Cornus officinalis*, and the content of iridoid glycosides in traditional wine processed *Cornus officinalis* was significantly reduced. The application of wine honey processed *Cornus officinalis* can increase the content of main iridoid glycosides to varying degrees, but the exact mechanism remains to be further clarified. In this study, DN rat model was induced by high-fat and high-sugar diet combined with intraperitoneal injection of streptozotocin (STZ). The therapeutic mechanism of *Cornus officinalis* before and after processing on DN and its effect on intestinal flora were investigated in order to provide a new experimental basis for the clinical application of *Cornus officinalis* and its processed products.

## Materials

### Animal

A total of 100 male SD rats, weighing 180–220 g, were provided by Liaoning Changsheng Biotechnology Co., Ltd., with the experimental animal production license number: SCXK (Liaoning) 2020-0001. According to the experimental animal management method of Liaoning University of Traditional Chinese Medicine, at room temperature to the standard feed, free drinking water, sub-cage (8 cages) adaptive feeding 1w for the test. All animals were raised in the SPF animal room of our laboratory and given humanitarian care according to the 3R (Reduction; Replacement; Refinement) principle. Authorized by the Ethics Committee of Liaoning University of Traditional Chinese Medicine.

### Drug

*Cornus officinalis* decoction pieces (place of origin: Xixia, Henan, harvest time: September 2021, identified by Professor Zhang Hui from Liaoning University of Traditional Chinese Medicine as the dried ripe pulp of *Cornus officinalis* Siebold & Zucc. Cornaceae.), yellow wine (Zhejiang Guyue Longshan Shaoxing Wine Co., Ltd., batch number: GB/T 13662), honey (wild mountain nectar, bee farm address: Linyi, Shandong, collection place: Yimeng Mountain Area), irbesartan dispersible tablets (Shuanghe Pharmaceutical Co., Ltd., national drug permit: 6935216804279).

### Reagents and instruments

STZ (Dalian Meilun Biotechnology Co., Ltd., Batch No. MB1227), high-fat diet (66.5% conventional feed, 10% lard, 20% sucrose, 2.5% cholesterol, 1% sodium cholate, 7-8 eggs, appropriate amount of dextrin), acetic acid, propionic acid, butyric acid, isobutyric acid, valeric acid, isovaleric acid, hexanoic acid, 4-methyl valeric acid (Shanghai Yuanye Biotechnology Co., Ltd., Batch No. 64-19-7, 79-09-4, 107-92-6, 79-31-2, 109-52-4, 503-74-2, 142-62-1, 646-07-1). Phosphoric acid (Tianjin Damao Chemical Reagent Factory, Batch No. 20220106), Trizol (Invitrogen life technologies, United States, Batch No. 15596026), horizontal cryopreservation box (Qingdao Haier Electric Appliance Co., Ltd., Batch No. DW-86W100), high-speed refrigerated centrifuge (SIGMA, Germany, Batch No. Sigma3k15), tissue dehydrator (Cherry Blossom, Japan, Batch No. VIP6 AI), embedding machine (Persjie, Batch No. BM450A). Tissue bleaching oven (Paisger, Batch No. PH60). Pathological section machine (Lecia, Batch No. Biocut), panoramic scanner (3DHISTECH, Batch No.: 3DHISTECH P250 FLASH), frozen section machine (Lecia, Batch No. CM 1900), gas chromatography-mass spectrometry, equipped with 7697A headspace sampler and MassHunter Workstation B.07.00 workstation (Agilent, United States, Model: 7890B-5977B). Capillary Column (30 m × 0.25 mm, 0.25 μm, Agilent, United States, Model: DB-WAX). DNA Extraction Kit (MP Biomedicals, United States, Model: FastDNA ^®^ Spin Kit for Soil), FastPfu Polymerase (TransGen, China, Model: FastPfu Polymerase), AxyPrep DNA Gel Extraction Kit (Axygen, United States, Model: Axygen Biosciences). Serum creatinine (S-Cr), blood urea nitrogen (BUN), 24 h urine microalbumin (mALB) detection kits (Shanghai Kexing Biotechnology Co., Ltd., batch numbers: F40108-A, F40289-A, F8560-A), Gold View dye, Evo M-MLV reverse transcription kit, SYBR ^®^ Green Pro Taq HS Premix kit (Aikeri Biological Engineering Co., Ltd., batch numbers: A2A1634, AG11711, AG11718). Rapid DNA-Seq Kit (Bioo Scientific, United States, Model: NEXTFLEX), Sequencing Kit (Illumina, United States, Model: MiSeq Reagent Kit v3/NovaSeq Reagent Kits), Microplate Reader (Biotek, United States, Model: BioTek ELx800), Vortex Mixer (Haimen Kylin-Bell Lab Instrument Manufacturing Co., Ltd., Model: QL-901). Grinder (MP, United States, Model: FastPrep-24 5G), Microfluorimeter (Promega, United States, Model: Quantus TM Fluorometer), Electrophoresis (Beijing Liuyi Instrument Factory, Model: DYY-6C), PCR (ABI, United States, Model: ABI GeneAmp ^®^ 9700), Sequencing (Illumina, United States, Model: Illumina Miseq).

## Methods

### Preparation of samples

Preparation of wine *Cornus officinalis*: take the pure *Cornus officinalis*, add an appropriate amount of excipients mixed, airtight, water-sparing stew for 8 h until the excipients are absorbed, take out, dried at 50°C for 4 h, each 600 g *Cornus officinalis* with excipients yellow wine 180 g. Preparation of wine honeyed *Cornus officinalis*: The *Cornus officinalis* meat was taken, mixed with appropriate amount of excipients, sealed, simmered in water for 6 h until the excipients were exhausted, taken out, dried at 50°C for 5 h, and 210 g (wine: honey = 30: 5) per 600 g of *Cornus officinalis* meat.

Each 600 g sample of *Cornus officinalis* meat, wine *Cornus officinalis* meat and wine honey *Cornus officinalis* meat were accurately weighed and boiled twice with water. The first time, 10 times the amount of water was added, boiled for 1.5 h, and the second time, 8 times the amount of water was added, boiled for 1.5 h. The filtrate was combined twice, and the concentration was concentrated to 0.28125 g·mL^−1^ by rotary evaporation. The crude product extract of *Cornus officinalis* meat was obtained, packed into glass bottles, sealed and stored at 4°C refrigerator for the experimental cycle.

### Modeling, grouping and administration

All rats were adaptively fed for 7 days, and 10 rats with abnormal coat color, spirit and stool were removed in time. The remaining rats were divided into normal group (*n* = 10) and DN group (*n* = 80) by random number table method. The rats in the blank group were fed with normal diet, and those in the DN group were fed with high-sugar and high-fat diet for 4 weeks. After fasting for 12 h, the DN rat model was induced by intraperitoneal injection of STZ 35 mg·kg^−1^ (prepared immediately, the solvent was citric acid-sodium citrate buffer). The rats in the blank group were given an equal volume of citric acid-sodium citrate buffer by single intraperitoneal injection. After 72 h, blood was taken from the tail vein and the blood glucose was measured by a blood glucose meter. The rats with fasting blood glucose (FBG) ≥ 16.7 mmol·L^−1^ and urine volume more than doubled were selected as DN rats. The blood glucose of 6 model rats was less than 16.7 mmol·L^−1^, and 4 rats died during the modeling process, all of which were eliminated. According to the random number table method, 70 successfully modeled rats were divided into DN model group, irbesartan group (1.35 mg·kg^−1^·d^−1^), *Cornus officinalis* group (281.25 mg·kg^−1^·d^−1^), wine Cornus officinalis group (281.25 mg·kg^−1^·d^−1^), wine honey Cornus officinalis group (281.25 mg·kg^−1^·d^−1^), auxiliary wine group (10 ml·kg^−1^·d^−1^) and auxiliary wine honey group (10 ml·kg^−1^·d^−1^), 10 rats in each group, according to the above drugs and doses, once a day for 6 weeks.

### Specimen retention and preservation

Observe the general state of rats. After 6 weeks of administration, blood glucose was measured by tail vein. Metabolic cages were used to collect 24 h urine of rats. During the period of fasting, water was not allowed. The urine volume of rats in each group was recorded. After centrifugation, the supernatant was taken and frozen at −20°C refrigerator for mAlb determination. Fecal samples of rats in each group were taken by the tail-raising reflex method in a sterile EP tube, frozen in liquid nitrogen, and frozen in a refrigerator at -80°C for the detection of intestinal flora diversity and short-chain fatty acids (SCFAs). The rats were fasted for 12 h before sampling, and the body weight of each group was weighed. After anesthesia with 20% urethane solution by intraperitoneal injection, 5 ml of blood was collected from the abdominal aorta, and the whole blood was collected by the procoagulant tube. After standing for 1 h, the serum was centrifuged (3500 r·min^−1^, 4°C, 15 min) and frozen for the determination of S-Cr and BUN. The left kidney was weighed and fixed in 4% paraformaldehyde solution, and the right kidney was quickly sub-packed in EP tube. After quick freezing in liquid nitrogen, it was frozen in -80°C ultra-low temperature refrigerator for RT-PCR determination of YKL-40, Wnt4, *β*-catenin, TGF-β1 mRNA expression.

### General state detection

Observe the reaction, mental status, diet, coat color, urine and stool of rats. The body weight, food intake and water intake of rats were monitored weekly.

### Biochemical index detection and kidney index calculation

The serum samples frozen at −80°C were thawed at 4°C refrigerator. The levels of S-Cr, BUN and mALB in rat serum samples were detected by ELISA kit. The experimental steps were carried out strictly according to the kit instructions. The kidney index (KI) is the ratio of kidney mass (mg) to body mass (g).

### Pathological observation of kidney tissue

The kidney tissues fixed with 4% paraformaldehyde for more than 48 h were dehydrated with different concentrations of alcohol, embedded in paraffin and sliced. After drying, xylene dewaxing, washing and HE staining, the morphology of kidney tissues in each group was observed under optical microscope.

### Expression of YKL-40, Wnt4, *β*-catenin and TGF-β1 mRNA

An appropriate amount of frozen kidney tissue samples were taken, homogenized, and total RNA was extracted in strict accordance with the instructions of the Trizol kit. RNA concentration was measured, and cDNA was synthesized by reverse transcription kit. According to SYBR Green PCR kit instructions, PCR reaction. PCR cycle reaction conditions: 95°C pre-denaturation 30 s, 95°C denaturation 15 s, 60°C annealing 1 min, 95°C extension 15 s, 40 cycles. The relative expression of the target gene in each sample was calculated by $2^{-∆∆CT}$ method (CT was the number of cycles). Each sample was repeated 3 times independently. The primer sequence is shown in Table 1.

**TABLE 1:** Genes and their corresponding primer sequences.

Primer name	Primer sequences	Primer length
YKL-40	Upstream: 5′-CTA​CTA​CGA​GAT​ATG​CGA​CTT-3′	21
	Downstream: 5′-CTT​GTT​CTT​CAG​GTA​CTT​CAC-3′	21
Wnt4	Upstream: 5′-ATA​CGC​CAT​CTC​TTC​AGC​AGG​T-3′	22
	Downstream: 5′-TCA​CAG​CCA​CAC​TTC​TCC​AGA​T-3′	22
β-catenin	Upstream: 5′-GTC​TGA​GGA​CAA​GCC​ACA​GGA​CTA​C-3′	25
	Downstream: 5′-AAT​GTC​CAG​TCC​GAG​ATC​AGC​A-3′	22
TGF-β_1_	Upstream: 5′-ATA​CGC​CTG​AGT​GGC​TGT​CTT​T-3′	22
	Downstream: 5′-AAA​GCC​CTG​TAT​TCC​GTC​TCC​T-3′	22
β-actin	Upstream: 5′-GGA​GAT​TAC​TGC​CCT​GGC​TCC​TA-3′	23
	Downstream: 5′-GAC​TCA​TCG​TAC​TCC​TGC​TTG​CTG-3′	24

### Detection of SCFAs

#### Headspace injection conditions

Headspace sampler sample bottle heating temperature: 80°C; quantitative ring heating temperature: 140°C; transmission line heating temperature: 160°C; GC cycle time: 30 min; heating time: 20 min; equilibrium time: 10 min; pressurization time: 0.15 min; injection time: 0.5 min.

#### Chromatographic and mass spectrometric conditions

Agilent DB-WAX (DB-1MS) chromatographic column (30 m × 0.25 mm, 0.25 μm); the injection method is not split; inlet temperature 250°C; ion source temperature 230°C; transmission line temperature 250°C, quadrupole temperature 150°C. The initial temperature of programmed heating was 60°C, and then increased to 120°C at a rate of 30°C·min^−1^.5°C·min^−1^–140°C for 1 min then heated to 150°C at 10°C·min^−1^ for 1 min; then heated to 160°C at 5°C·min^−1^ for 1 min; finally, the temperature was raised to 230°C at a rate of 35°C·min^−1^, and then maintained at 230°C for 5 min. The carrier gas was helium at a flow rate of 1.0 ml·min^−1^.

Electron impact ionization (EI), 70 ev electron energy, solvent delay 4.5 min, full scan (SCAN) and selected ion monitoring (SIM) modes, scan range 30–200 m·z^−1^.

#### Preparation of standard solution

The standard substances of acetic acid, propionic acid, butyric acid, isobutyric acid, isovaleric acid, valeric acid and hexanoic acid were accurately weighed. The mixed standard solution was prepared with ultrapure water, and the mixed working solution with different concentrations was obtained by gradient dilution. Acetic acid 36.69 μg·mL^−1^, propionic acid 1.01 μg·mL^−1^, isobutyric acid 1.50 μg·mL^−1^, butyric acid 16.24 μg·mL^−1^, isovaleric acid 1.14 μg·mL^−1^, valeric acid 3.00 μg·mL^−1^, hexanoic acid 1.10 μg·mL^−1^ were prepared in the stock solution.

#### Determination of short chain fatty acid content in feces of rats in each group

A total of 100 mg sterile feces of rats in each group at the same time point were weighed accurately and placed in a 20 ml headspace injection bottle. Add 50 μL 0.2% H3PO4 solution, containing 4-methylpentanoic acid internal standard solution (0.668 mg·mL^−1^), fully mixed, quickly sealed, and detected in GC-MS instrument. In order to ensure the true repeatability of the experimental results, the same sample needs to be divided into 6 parts, and the injection is completed one by one. The corresponding SCFAs peak area is calculated, and the concentration of each short-chain fatty acid in the supernatant is calculated according to the standard curve.

### Detection of intestinal flora in rats by 16S rRNA high-throughput sequencing

#### DNA extraction and PCR amplification

The total DNA in feces was extracted according to the DNA extraction kit, and the quality of DNA extraction was detected by 1% agarose gel electrophoresis. NanoDrop2000 was used to determine DNA concentration and purity. The V3-V4 variable region of 16S rRNA gene was amplified by PCR with primers 338F (5′-ACT​CCT​ACG​GGA​GGG​CAG​CAG-3′) and 806R (5′-GGACTACHVGGGTWTCTAAT-3′). The amplification procedure was as follows: pre-denaturation at 95°C for 3 min, 27 cycles (denaturation at 95°C for 30s, annealing at 55°C for 30 s, extension at 72°C for 30 s), then stable extension at 72°C for 10min, and finally stored at 4°C. Each sample was repeated three times.

#### Illumina Miseq sequencing

The PCR products of the same sample were mixed and recovered using 2% agarose gel. The recovered products were purified using the AxyPrep DNA Gel Extraction Kit, examined by 2% agarose gel electrophoresis, and quantified using the Quantus TM Fluorometer. Using the NEXTFLEX Rapid DNA-Seq Kit to build the library linker first; using magnetic beads for screening to remove the joint self-connected segment; the library template was enriched by PCR amplification. Finally, the PCR products were recovered by magnetic beads to obtain the library. Sequencing was performed using Illumina‘s 61 Miseq PE300 platform.

#### Data processing

Fastp (Chen et al., 2018) software was used to perform quality control on the original sequencing sequence, and FLASH (Magoč and Salzberg, 2011) software was used for splicing: 1) Filter the bases with a tail mass value of less than 20 in the reads, and set a 50 bp window. If the average mass value in the window is less than 20, the back-end bases are removed from the window, and the reads below 50 bp after quality control are filtered to remove the reads containing N bases; 2) According to the overlap relationship between PE reads, the paired reads were merged into a sequence with a minimum overlap length of 10 bp. 3) The maximum allowed mismatch ratio of overlap region of concatenated sequences is 0.2, screening non-conforming sequences; 4) According to the sequence of both ends of the barcode and primers to distinguish between samples, and adjust the direction of the sequence, barcode allows the mismatch is 0, the maximum primer mismatch is 2.

Using UPARSE (Edgar, 2013) software, OTU clustering was performed on the sequence according to 97% similarity and chimeras were removed. RDP classifier (Stackebrandt and Goebel, 1994; Wang et al., 2007) was used to annotate the species classification of each sequence. The Silva 16S rRNA database was compared, and the comparison threshold was set to 70%.

### Statistical methods

SPSS 20.0 software was used for statistical analysis. Graphpad Prism 8.0 was used for plotting. The data were expressed as mean ± standard deviation ( $\bar{x}\pm s$ ). The *t* test was used for pairwise comparison, and one-way analysis of variance was used for comparison among multiple groups. *p* < 0.05 indicated that the difference was statistically significant.

## Results

### Changes of general signs in rats

During the experiment, the rats in the blank group grew well, the fur was bright, the activity was large, the reaction was agile, the eating and excretion were normal, and the body weight gradually increased. The rats in the DN model group, auxiliary wine group, and the auxiliary wine honey group grew significantly slower, had less activity, and drank more, ate more, and urinated more. In the later stage of the experiment, the rats in the DN model group, auxiliary wine group, and the auxiliary wine honey group showed obvious slow response, dull fur, and obvious weight loss. Compared with the DN model group, the rats in the irbesartan group and the wine honey Cornus officinalis group were significantly improved in the above aspects. In the later stage of the experiment, the body weight of the DN model group was significantly lower than that of the other groups.

### Biochemical indicators level detection

**FIGURE 1:**
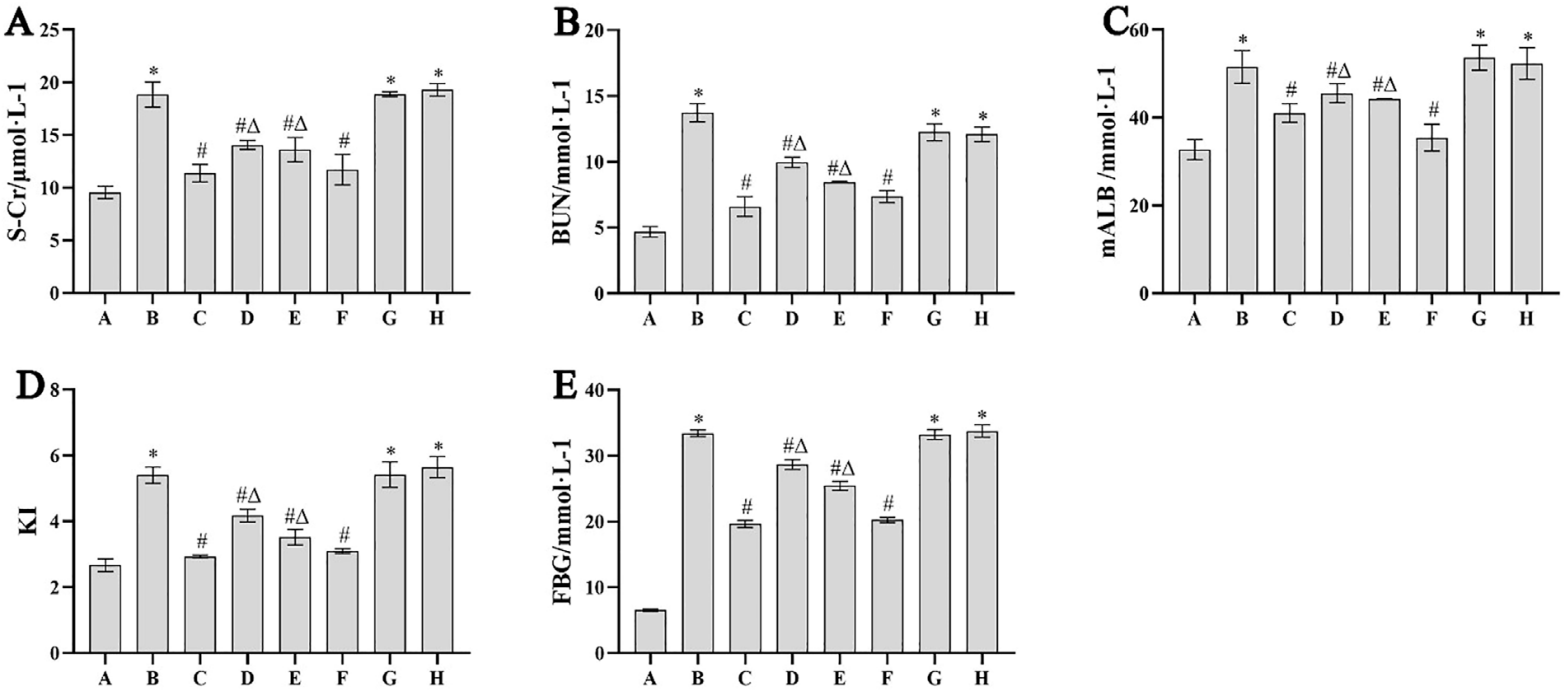
Comparison of S-Cr **(A)**, BUN **(B)**, mALB **(C)**, KI **(D)** and FBG **(E)** in each group (*n* = 10, $\bar{x}\pm s$ ). Note: ① A. Blank group B. DN model group C. Irbesartan group D. *Cornus officinalis* group E. Wine Cornus officinalis group F. Wine honey Cornus officinalis group G. Auxiliary wine group H. Auxiliary wine honey group. ② Compared with the blank group ^*^ *p* < 0.05; compared with DN model group ^#^ *p* < 0.05; compared with the wine honey group ^Δ^ *P* < 0.05.

Compared with the blank group, S-Cr, BUN, mALB, KI and FBG in DN model group, auxiliary wine group and auxiliary wine honey group were significantly increased (*p* < 0.05), and the difference was statistically significant. Compared with the DN model group, the S-Cr, BUN, mALB, KI and FBG of the rats in the irbesartan group, the *Cornus officinalis* group, the wine Cornus officinalis group and the wine honey Cornus officinalis group were significantly decreased (*p* < 0.05). The levels of S-Cr, BUN, mALB, KI and FBG in the wine honey Cornus officinalis group were significantly lower than those in the *Cornus officinalis* group and the wine Cornus officinalis group (*p* < 0.05). See Figure 1.

### Pathological examination of renal tissue of rats in each group

**FIGURE 2:**
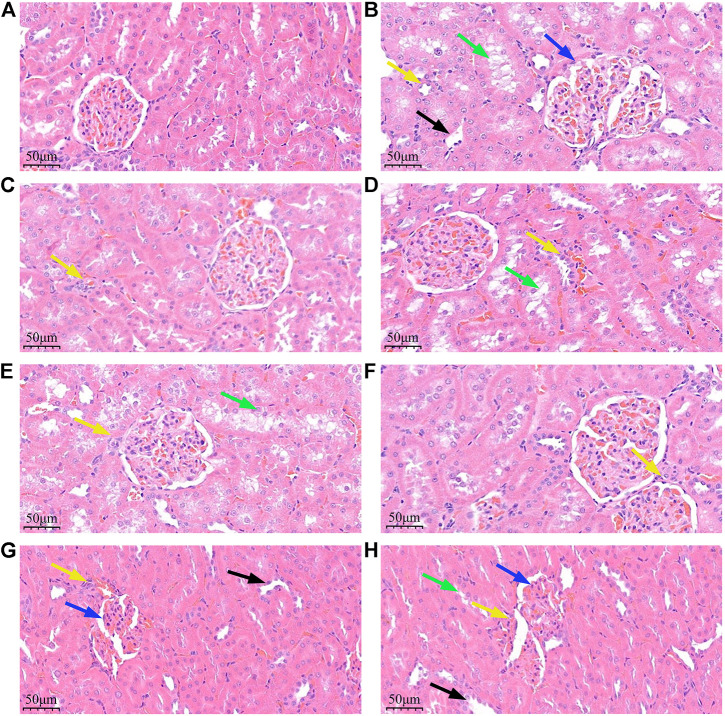
Effects of *Cornus officinalis* and its processed products on renal histopathology in DN rats (HE, ×400). Note: ① **(A)**. Blank group **(B)**. DN model group **(C)**. Irbesartan group **(D)**. Cornus officinalis group E. Wine Cornus officinalis group **(F)**. Wine honey Cornus officinalis group **(G)**. Auxiliary wine group **(H)**. Auxiliary wine honey group. ② Blue arrows indicate glomerular deformation, black arrows indicate renal interstitial edema, green arrows indicate renal tubular degeneration and atrophy, and yellow arrows indicate interstitial inflammatory cell infiltration.

The pathological examination results showed that the blank group rats had normal renal tissue morphology, regular glomerular shape, no hypertrophy or atrophy, normal arrangement of renal tubular epithelial cells, and normal renal interstitial structure. There were obvious pathological changes of DN in the kidney tissue of rats in the DN model group, the auxiliary wine group and the auxiliary wine honey group. The renal tissue structure was disordered, glomerular hypertrophy, mesangial cell proliferation, nodular sclerosis was clearly visible, renal tubular degeneration and atrophy. A large number of vacuoles and swollen cells were seen in the renal tubular epithelial cells. Obvious vacuolar degeneration, partial atrophy, obvious edema of renal, and a large number of inflammatory cell infiltration. Compared with the DN model group, the above pathological changes of renal tissue in the *Cornus officinalis* group, the wine Cornus officinalis group, the wine honey Cornus officinalis group and the irbesartan group were significantly improved, and the glomerular nodule sclerosis and renal interstitial edema were reduced. The most obvious was the degree of pathological inflammatory infiltration, glomerular atrophy, and mesangial proliferation in the wine honey Cornus officinalis group. See Figure 2.

### Expression of YKL-40, Wnt4, *β*-catenin and TGF-*β*_1_ mRNA in renal tissue of rats

**FIGURE 3:**
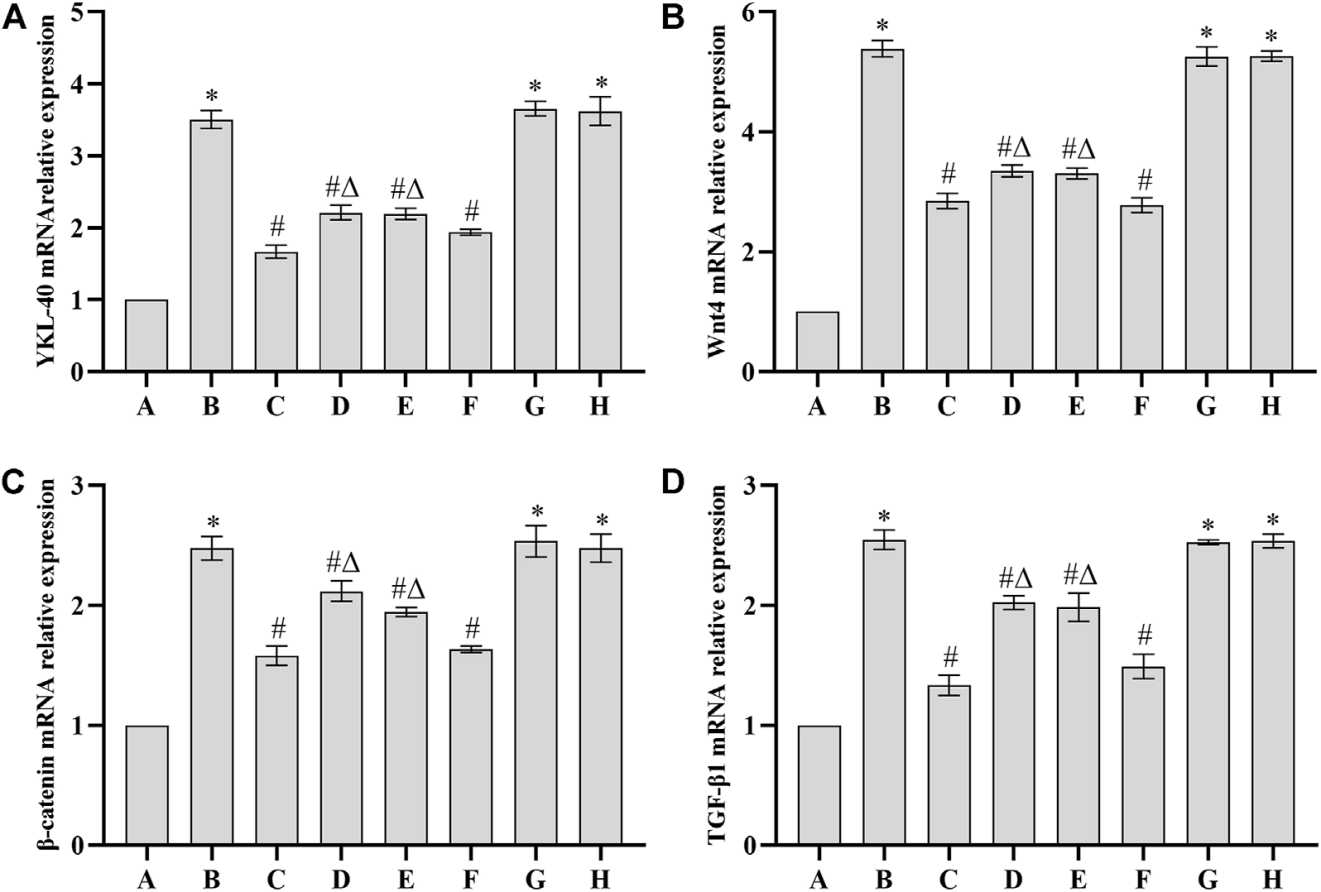
Comparison of mRNA expression of YKL-40 **(A)**, Wnt4 **(B)**, *β*-catenin **(C)** and TGF-β1 **(D)** in each group (*n* = 10, $\bar{x}\pm s$ ). Note: A. Blank group B. DN model group C. Irbesartan group D. *Cornus officinalis* group E. Wine Cornus officinalis group F. Wine honey Cornus officinalis group G. Auxiliary wine group H. Auxiliary wine honey group.

Compared with the blank group, the expression of YKL-40, Wnt4, *β*-catenin and TGF-*β*_1_ mRNA in renal tissue of DN model group, auxiliary wine group and auxiliary wine honey group was significantly increased (*p* < 0.05). Compared with DN model group, the expression of YKL-40, Wnt4, *β*-catenin and TGF-*β*_1_ mRNA in renal tissue of *Cornus officinalis* group, wine Cornus officinalis group and wine honey Cornus officinalis group was significantly decreased (*p* < 0.05). Compared with the wine honey Cornus officinalis group, the expression of YKL-40, Wnt4, *β*-catenin and TGF-*β*_1_ mRNA in the kidney tissue of the *Cornus officinalis* group and the wine Cornus officinalis group was significantly different (*p* < 0.05). See Figure 3.

### Results of SCFAs in rats

Compared with the blank group, the contents of seven short-chain fatty acids in the fresh feces of the DN model group, the auxiliary wine group, and the auxiliary wine honey group were significantly reduced (*p* < 0.05). After drug intervention, it was found that the irbesartan group, the wine Cornus officinalis group, and the wine honey Cornus officinalis group had a significant callback effect on the content of SCFAs in fresh feces. In addition to the significant increase in butyric acid in fresh feces, the *Cornus officinalis* group also had a wide callback effect on the other six SCFAs (*p* < 0.05). Although the range and intensity of SCFAs in each sample were slightly different in each administration group, except for butyric acid in each administration group. They all had significant up-regulation (*p* < 0.05). In short, the wine honey Cornus officinalis group had the best regulation effect on the content of 7 kinds of SCFAs (Table 2).

**TABLE 2:** Intestinal SCFAs content of rats in each group (*n* = 6, $\bar{x}\pm s$ , μg/g).

Peer group	Acetic acid	Propionic acid	Isobutyric acid	Butyric acid	Isovaleric acid	Valeric acid	Caproic acid
A	1261.57 ± 41.70	111.57 ± 2.56	120.27 ± 4.86	69.97 ± 9.39	4.50 ± 0.98	37.63 ± 4.76	49.91 ± 2.32
B	800.85 ± 71.23^*^	67.28 ± 2.62^*^	68.03 ± 7.28^*^	35.72 ± 8.66^*^	1.75 ± 0.48^*^	23.06 ± 7.22^*^	13.68 ± 1.29^*^
C	1162.64 ± 80.62^#^	103.57 ± 6.92^#^	107.70 ± 4.55^#^	65.28 ± 5.91^#^	3.90 ± 0.84^#^	35.03 ± 7.72^#^	46.47 ± 0.78^#^
D	901.44 ± 48.42^#Δ^	94.64 ± 2.66^#Δ^	100.11 ± 4.39^#^	38.53 ± 5.85^Δ^	2.86 ± 0.47^#Δ^	29.30 ± 2.64^#^	30.22 ± 1.97^#Δ^
E	1017.26 ± 73.56^#Δ^	89.32 ± 3.27^#Δ^	92.08 ± 5.83^#Δ^	53.20 ± 7.39^#Δ^	3.00 ± 0.55^#Δ^	32.97 ± 3.60^#^	28.42 ± 0.70^#Δ^
F	1126.45 ± 51.19^#^	108.77 ± 4.45^#^	106.04 ± 3.48^#^	62.63 ± 7.71^#^	3.84 ± 0.84^#^	34.75 ± 3.58^#^	46.60 ± 2.09^#^
G	799.45 ± 74.81^*^	67.25 ± 5.24^*^	69.81 ± 8.90^*^	35.84 ± 2.56^*^	2.29 ± 0.66^*^	22.54 ± 2.26^*^	16.47 ± 0.52^*^
H	808.69 ± 81.10^*^	69.20 ± 3.10^*^	71.92 ± 9.50^*^	33.45 ± 2.27^*^	2.38 ± 0.60^*^	22.36 ± 7.34^*^	17.76 ± 1.06^*^

Note: ① A. Blank group B. DN, model group C. Irbesartan group D. *Cornus officinalis* group E. Wine Cornus officinalis group F. Wine honey Cornus officinalis group G. Auxiliary wine group H. Auxiliary wine honey group. ② Compared with normal group ^*^ *p* < 0.05; compared with the model group ^#^ *p* < 0.05; compared with the wine honey group ^Δ^ *P* < 0.05.

### Analysis of differences in intestinal flora of rats at different classification levels

#### OUT analysis of intergroup species

**FIGURE 4:**
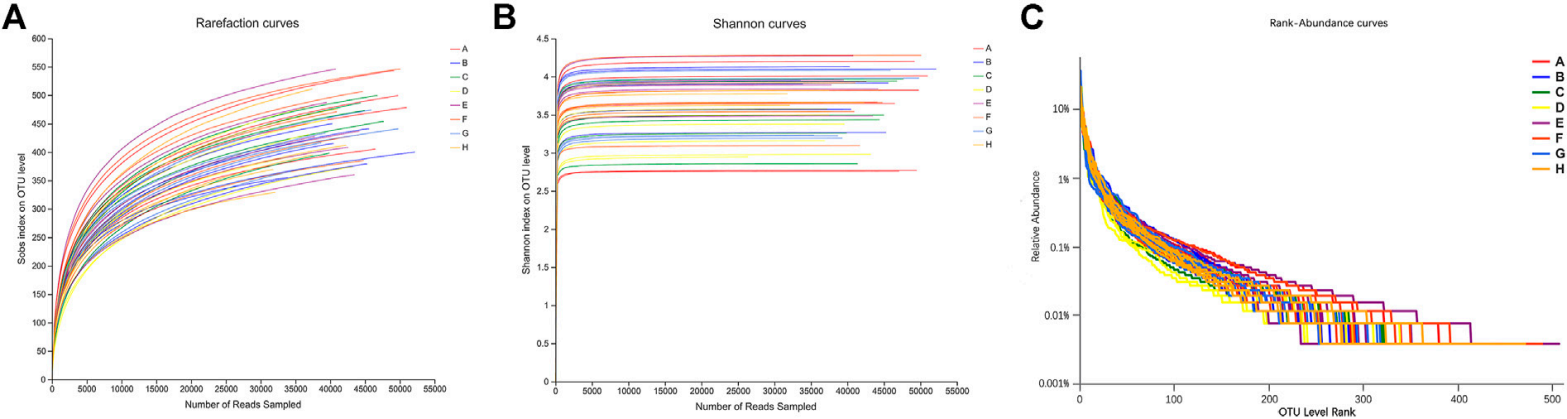
Sequencing Dilution Curve **(A)**, Shannon-Wiener Curve **(B)** and Rank-Abundance Curve **(C)** of Intestinal Microflora in Fecal Samples of Rats in Each Group. Note: A. Blank group B. DN model group C. Irbesartan group D. *Cornus officinalis* group E. Wine Cornus officinalis group F. Wine honey Cornus officinalis group G. Auxiliary wine group H. Auxiliary wine honey group.

According to the number of OTUs when the diversity index of the sample is sobs at different sequencing depths, the dilution curve (Figure 4) is drawn. It can be seen that the curve gradually flattens out when the sample is around 30000Reads, indicating that the amount of sequencing data is reasonable. More data will only produce a small number of new species; according to the number of OTUs with Shannon-Wiener diversity index at different sequencing depths, the curve was drawn (Figure 4). It can be seen that the curve gradually tends to be flat when the sample is around 4000Reads, indicating that the amount of sequencing data is large enough to reflect the vast majority of microbial diversity information in the sample.

**FIGURE 5:**
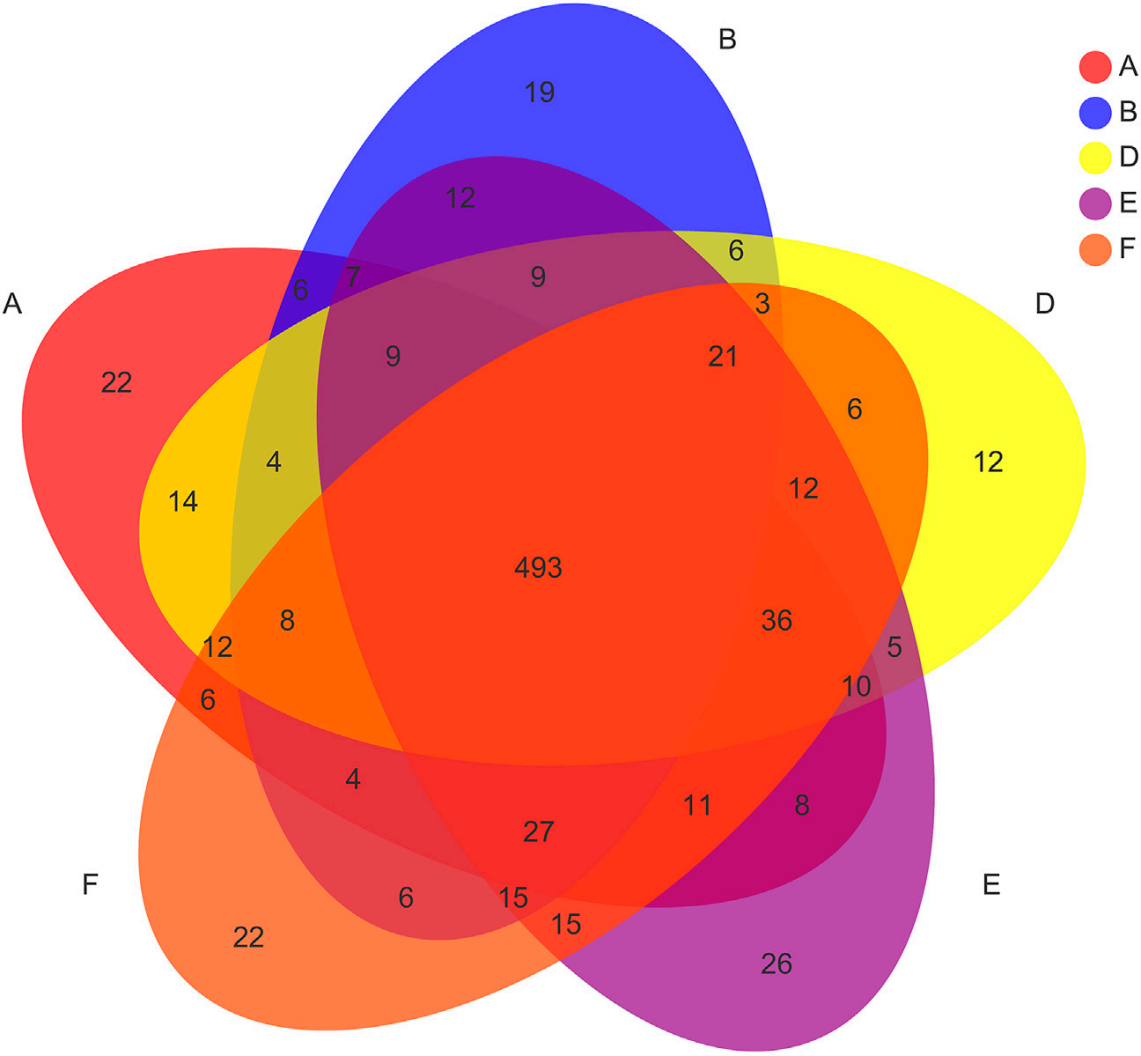
Veen plot of OUT distribution in different treatment groups. Note: A. Blank group B. DN model group C. Irbesartan group D. *Cornus officinalis* group E. Wine Cornus officinalis group F. Wine honey Cornus officinalis group G. Auxiliary wine group H. Auxiliary wine honey group.

The Venn diagram was used to count the number of common and unique OTUs in 5 groups of samples, which could directly show the similarity and overlap of OTU composition in different environmental samples. The intestinal flora OTU of each group (Figure 5). There were 22 unique OTUs in the blank group, 19 unique OTUs in the DN model group, 12 unique OTUs in the *Cornus officinalis* group, 26 unique OTUs in the wine Cornus officinalis group, and 22 unique OTUs in the wine honey Cornus officinalis group. There were 493 OTUs shared by the five groups.

#### Alpha diversity analysis of intergroup species

Alpha diversity reflects the species diversity within a single sample. It is measured by community abundance index and community diversity index. The community abundance index includes Chao1 and Ace. The larger the index value, the higher the richness of the community. The community diversity index includes Shannon and Simpson. The larger the index value, the higher the species diversity of the sample. The results showed that the richness (Chao1 and Ace) and diversity (Shannon and Simpson) of intestinal flora in the feces of DN model rats were lower than those in the normal group (*p* < 0.05). The richness (Chao1 and Ace) and diversity (Shannon and Simpson) of intestinal flora in the feces of irbesartan group, *Cornus officinalis* group, wine Cornus officinalis group and wine honey Cornus officinalis group were significantly different from those in the DN model group (*p* < 0.05), close to the blank group. See Table 3.

**TABLE 3:** Effects of Cornus officinalis before and after processing on Alpha diversity index of intestinal flora in DN rats (*n* = 6, $\bar{x}\pm s$ ).

Peer group	Shannon	Simpson	Ace	Chao
A	4.34 ± 0.26	0.16 ± 0.02	595.16 ± 22.19	554.79 ± 25.67
B	2.73 ± 0.37*	0.06 ± 0.02*	361.62 ± 10.08*	325.24 ± 16.60*
C	4.18 ± 0.43#	0.11 ± 0.02#	518.78 ± 22.03#	519.91 ± 26.76#
D	3.78 ± 0.20#	0.08 ± 0.03	417.43 ± 37.08#	455.18 ± 31.12#
E	3.84 ± 0.30#	0.07 ± 0.04	426.09 ± 47.90#	472.91 ± 49.46#
F	3.84 ± 0.33#	0.10 ± 0.02#	421.74 ± 49.67#	516.64 ± 26.65#
G	2.73 ± 0.16*	0.05 ± 0.02*	350.35 ± 27.10*	321.29 ± 13.12*
H	2.75 ± 0.20*	0.06 ± 0.01*	348.90 ± 16.96*	327.96 ± 12.23*

Note: ① A. Blank group B. DN, model group C. Irbesartan group D. *Cornus officinalis* group E. Wine Cornus officinalis group F. Wine honey Cornus officinalis group G. Auxiliary wine group H. Auxiliary wine honey group. ② Compared with normal group ^*^ *p* < 0.05; compared with model group ^#^ *p* < 0.05.

#### Beat diversity analysis of intergroup species

**FIGURE 6:**
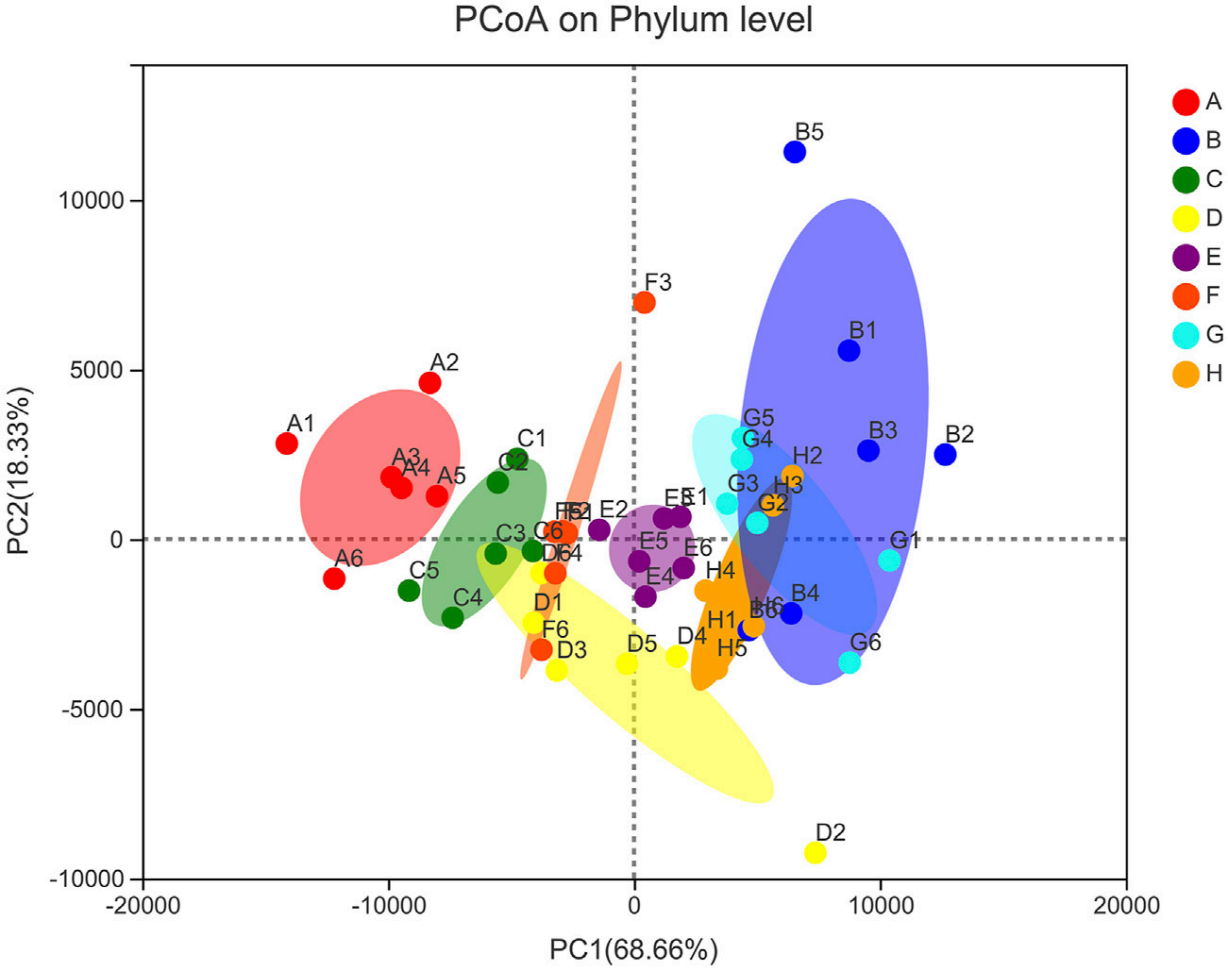
Distribution of PCoA scores of intestinal flora in rats of each group. Note: A. Blank group B. DN model group C. Irbesartan group D. *Cornus officinalis* group E. Wine Cornus officinalis group F. Wine honey Cornus officinalis group G. Auxiliary wine group H. Auxiliary wine honey group.

The principal coordinate analysis (PCoA) is shown in Figure 6, and the results show that the percentages of PCo1 and PCo2 explaining the overall variance are 68.66% and 18.33%, respectively. The blank group and the DN model group were completely separated. Compared with the blank group, the flora structure of the DN model group was significantly different. The sample points of *Cornus officinalis* group, wine Cornus officinalis group and wine honey Cornus officinalis group were closer than those of DN model group, and tended to be blank group, indicating that Cornus officinalis could affect the intestinal flora composition of DN rats. The sample points of auxiliary wine and auxiliary wine honey group were closer to the DN model group, indicating that auxiliary wine and auxiliary wine honey could not affect the intestinal flora composition of DN rats. Intestinal flora changed significantly in DN state, and Cornus officinalis could reverse this change before and after processing. The effect of wine honey Cornus officinalis was better than that of Cornus officinalis and wine Cornus officinalis.

#### Phylum level analysis of intestinal flora structure

**FIGURE 7:**
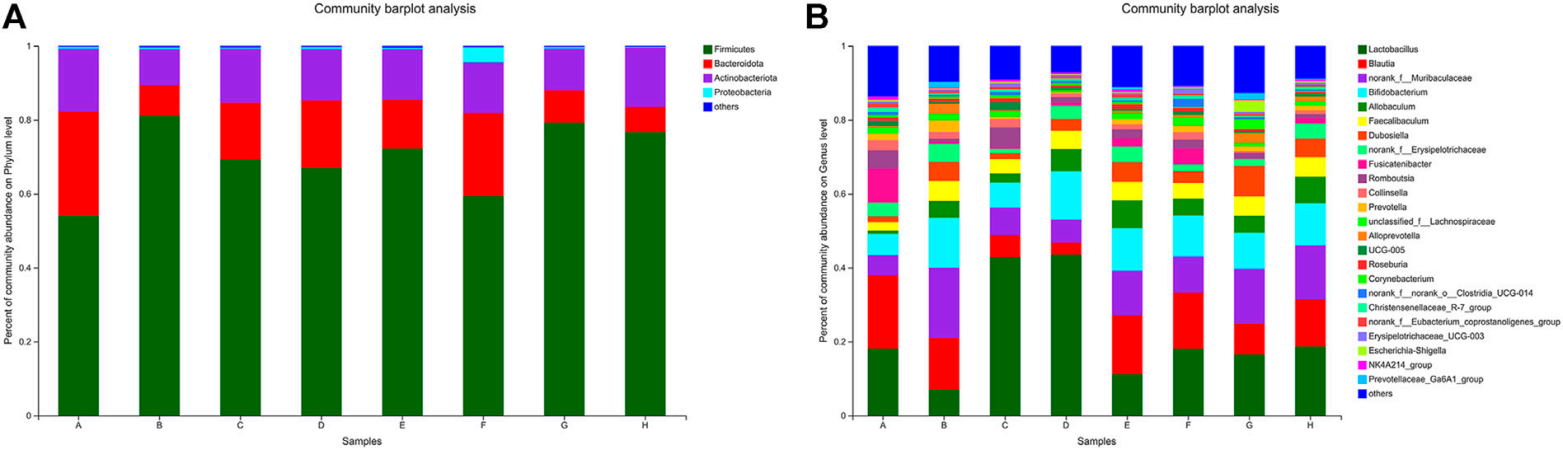
**(A)** Genus **(B)** Taxonomic composition analysis at taxonomic level. Note: A. Blank group B. DN model group C. Irbesartan group D. *Cornus officinalis* group E. Wine Cornus officinalis group F. Wine honey Cornus officinalis group G. Auxiliary wine group H. Auxiliary wine honey group.

At the level of phylum classification, the composition of the flora in each group of samples is shown in Figure 7A. In the blank group, DN model group and each administration group, the main phylum classification is Firmicutes, Bacteroidetes, Actinobacteriota and Proteobacteria. Among them, Firmicutes has the highest relative abundance, followed by Bacteroidota, and Proteobacteria has the lowest relative abundance. At the genus classification level, see Figure 7B, blank group, DN model group, the relative abundance of each drug group is higher for *Lactobacillus*.

#### Screening of differential bacteria

**FIGURE 8:**
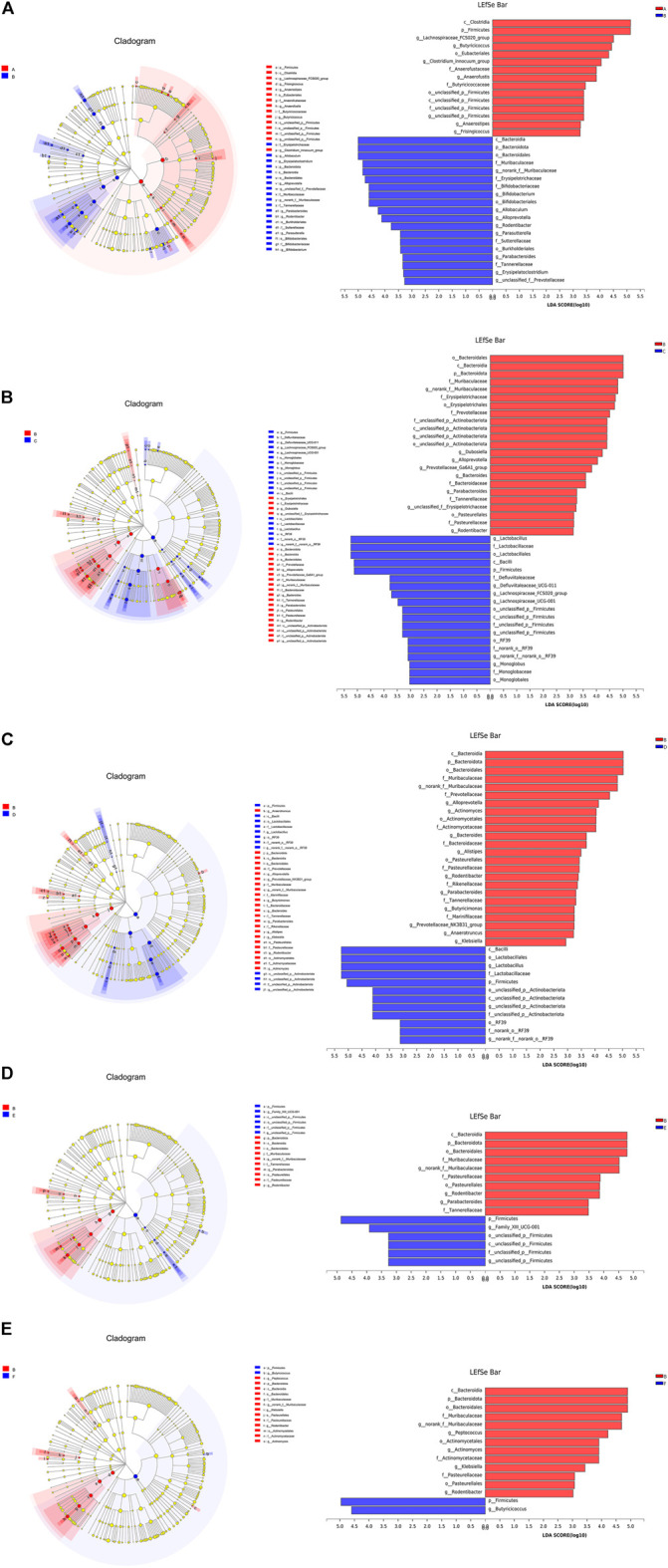
(Continued).

Linear discriminant analysis was performed with LEfSe software according to the taxonomic composition of the samples under different grouping conditions to find out the communities or species that had significant differences in sample division. LEfSe analysis has been widely used to find biomarkers with significant differences in abundance between two groups. LEfSe analysis results between each group and the model group are shown in Figure 8.

Compared with the blank group, the DN model group rats’ feces in the door, class, order, family, genus classification levels were found on the differential flora. The c_Bacteroidia, p_Bacteroidia and o_Bacteroidia in the feces of the DN model group were significantly higher than those in the blank group, while the dominant bacteria in the feces of the blank group were c_Clostridia, p_Firmicutes, g_Lachnospiracea_FCS020_group, g_Butyricicoccus, etc.

Compared with the DN model group, the g_*Lactobacillus*, f_Lactobacillaceae and o_Lactobacillales in the feces of the irbesartan group were significantly larger than those in the DN model group, while the dominant bacteria in the feces of the DN model group were o_Bacteroidales, c_Bacteroidia, p_Bacteroidota, f_Muribaculaceae, etc.

Compared with the DN model group, c_Bacilli, o_Lactobacillales, g_*Lactobacillus* and f_Lactobacillaceae in the feces of rats in the *Cornus officinalis* group were significantly higher than those in the DN model group. The dominant bacteria in feces of DN model group were c_Bacteroidia, p_Bacteroidota, o_Bacteroidales, f_Muribaculaceae and so on.

Compared with the DN model group, the p_Firmicutes, g_Family_XIII_p_UDG-001, o_unclassified_p_Firmicutes, c_unclassified_p_Firmicutes in the feces of the rats in the wine cornus group were significantly higher than those in the DN model group; the dominant bacteria in the feces of the DN model group were c_Bacteroidia, p_Bacteroidia, o_Bacteroidales, f_Muribaculaceae, etc.

Compared with the DN model group, the p_Firmicutes and g_Butyricicoccus in the feces of the rats in the wine honey Cornus officinalis group were significantly larger than those in the model group. The dominant bacteria in feces of DN model group were c_Bacteroidia, p_Bacteroidota, o_Bacteroidales, f_Muribaculaceae, g_norank_f_Muribaculaceae and so on.

## Discussions

Kidney is the most common organ involved in diabetes, DN will eventually develop into end-stage renal disease, affecting the prognosis of patients (Liu et al., 2022). The main clinical pathological changes of DN are glomerular sclerosis and tubulointerstitial fibrosis, and the main manifestations of renal interstitial fibrosis are renal parenchymal cell injury and a large number of extracellular matrix deposition (Guo et al., 2022). Studies have shown that *Cornus officinalis* can inhibit renal tubulointerstitial injury in diabetic rats, improve endoplasmic reticulum stress in mesangial cells, reduce mitochondrial autophagy, reduce renal pathological damage, improve renal fibrosis in DN rats, and inhibit cell proliferation (Lu, 2017; Zhao et al., 2018; Chen et al., 2020). In order to study the effect of *Cornus officinalis* and its processed products on the treatment of DN, this study replicated the DN rat model of streptozotocin model, and treated the DN rats with 281.25 mg/kg *Cornus officinalis*, 281.25 mg/kg wine Cornus officinalis and 281.25 mg/kg wine honey Cornus officinalis. The results showed that compared with the normal control group, the S-Cr, BUN, mALB and FBG in the model group were significantly increased, while the above indexes were decreased after the intervention of *Cornus officinalis* and its different processed products, and the decrease was obvious in the honey wine Cornus officinalis group.

The main pathological changes of DN are glomerulosclerosis and tubulointerstitial fibrosis (Zheng et al., 2022). The results of renal tissue section staining in this experiment suggest that the structure of renal tissue is disordered, glomerular hypertrophy, mesangial cell proliferation, nodular sclerosis is clearly visible, renal tubular degeneration and atrophy, a large number of vacuoles in the cytoplasm of renal tubular epithelial cells and swelling cells are obviously vacuolar degeneration, partial atrophy, renal interstitial edema and other obvious changes. The detection of the left kidney index of rats in each group also found that the kidney index of the model group was significantly increased, suggesting that the composition of the kidney tissue of the rats was changed. After treatment with each administration group, the left kidney index of the rats decreased, and the wine honey Cornus officinalis group decreased most significantly. The above studies showed that after the treatment of *Cornus officinalis* and its processed products, the pathological changes such as renal fibrosis and renal tubular sclerosis in DN rats were alleviated, indicating that *Cornus officinalis* can effectively improve the renal structure and clinical manifestations of DN rats, with the best effect of wine honey Cornus officinalis.

The pathogenesis of DN is complex, and Wnt signaling pathway may also be involved in the occurrence and development of kidney disease by regulating the proliferation and apoptosis of glomerular intrinsic cells (Guo and Xu, 2022). The results of this study showed that the expression of YKL-40, Wnt4, *β*-catenin and TGF-β1 mRNA in renal tissue of DN rats was significantly increased, suggesting that Wnt/β-catenin signaling pathway was activated in renal tissue of DN rats. The expression of YKL-40, Wnt4, *β*-catenin and TGF-β1 mRNA was significantly decreased after intervention in each administration group, suggesting that *Cornus officinalis* and its processed products may inhibit the development of DN and reduce the pathological changes of renal tissue by inhibiting Wnt/β-catenin signaling pathway.

After SCFAs are produced in the intestine and excreted through feces, they can quickly enter the systemic circulatory system and play a very important role in regulating the body’s normal physiological functions (Postler and Ghosh, 2017). It has been reported that propionic acid and butyric acid in SCFAs are important signal transduction molecules that bind to G protein-coupled receptors and inhibit histone deacetylase activity (McNabney and Henagan, 2017). Compared with the blank group, the content of various short-chain fatty acids in the DN model group was down-regulated, indicating that DN would reduce the content of short-chain fatty acids in rats. After administration, the range and intensity of regulation of each administration group were slightly different, but each administration group except butyric acid, were significantly up-regulated, indicating that each administration group had a better effect to improve the content of short-chain fatty acids in rats. The wine honeyed Cornus officinalis group had the best regulating effect on the content of short chain fatty acids in rats.

 Theresults of intestinal flora diversity test showed that compared with the normal group, the flora in the fecal samples of the DN model group changed significantly from phylum to genus level. Although the structure of intestinal flora in fecal samples is similar, the composition ratio between different phyla and genera is quite different. In the fecal samples, the relative abundance of Firmicutes was the highest in the phylum classification level, followed by Bacteroidetes, and the rest also included Actinobacteria and Proteobacteria. At the level of genus classification, *Lactobacillus* had higher relative abundance. In mice and humans, Firmicutes and Bacteroidetes are major components of the level of classification of intestinal flora. Studies have shown that the imbalance of intestinal flora can cause obesity (Qiu et al., 2022), and is related to the proportion of Firmicutes and Bacteroidetes. The higher the proportion, the more obvious the symptoms of obesity (Cheng et al., 2021); studies have also found that the decrease in the ratio is associated with improved glucose levels, reduced fat accumulation, and reduced body weight (Zeng et al., 2016). In the fecal samples of rats in this experiment, compared with the blank group, the abundance of Firmicutes in the DN model group increased, the abundance of Bacteroidetes decreased, and the ratio increased. *Lactobacillus* is a microorganism that lives in the body and benefits the health of the host. It can participate in regulating immunity and insulin resistance, thereby further regulating lipid and glucose metabolism (Yin et al., 2022); studies have also shown that increasing the proportion of *Lactobacillus* can reduce the risk of infection in patients with type 2 diabetes and has a protective effect on blood glucose (Zhou and Xu, 2021). In the fresh fecal samples of rats in this experiment, *Lactobacillus* in the DN model group showed a downward trend.

In summary, *Cornus officinalis* can effectively reduce DN rats 24-h urinary albumin, reduce renal pathological damage. *Cornus officinalis* may play a role in renal protection by anti-oxidation and inhibition of Wnt/β-catenin signaling pathway, regulating the content of some short-chain fatty acid-producing bacteria to increase the content of short-chain fatty acids in the intestine, and playing the role of energy supply in the body, which is conducive to healthy recovery, increasing beneficial bacteria and reducing pathogenic bacteria. The effect of wine honey Cornus officinalis is the best, which is expected to become a therapeutic drug for DN. The results of this experiment can provide a scientific basis for the rational development and utilization of Corni Fructus after processing.

## Data Availability

The datasets presented in this study can be found in online repositories. The names of the repository/repositories and accession number(s) can be found in the article/Supplementary material.

## Abbreviations

BUN, blood urea nitrogen; DN, Diabetic nephropathy; EI, electron impact ionization; FBG, fasting blood glucose; GC-MS, Gas Chromatography-Mass Spectrometer; HE, Hematoxylin-eosin; KI, kidney index; LDA, linear discriminant analysis; LEfSe, LDA Effect Size; mALB, 24 h urine microalbumin; PCoA, principal coordinate analysis; SCAN, full scanning mode; SCFAs, short chain fatty acids; SIM, selective ion monitoring mode; STZ, streptozocin; S-Cr, Serum creatinine.
